# California

**Published:** 1896-09

**Authors:** 


### CONTROL WORK.

*California.* Veterinarian Creely, of San Francisco, is doing valuable work as inspector for his city in arousing public opinion from its lethargy and demanding that legislation shall at once be inaugurated to afford their people greater safeguards from an impure and unwholesome milk- and meat-supply.

Oakland will have a milk ordinance that will be a great boon to her people. Veterinarian Pierce is conducting educational autopsies on tuberculin condemned animals, selecting those whose appearance would rather discourage the belief that they were diseased. One herd revealed 50 sound ones in 120. Within four months 18 had died from tuberculosis. Another herd of 143, where 20 had died, the owner continued to make butter for the public markets without sterilizing the milk or cream. These two herds were on Grizzly Island.

"Peddlers of Poison" is the headline of the *News*, of San José, in speaking of dispensers of milk from tuberculous herds. Veterinarian Spencer reported 892 cattle examined; 225 condemned from March 20th to July 1, 1896. In July 421 tested; 150 reacted. A similar percentage throughout the counties would show 1675 diseased in a total of 8377 kept as recorded by assessors' books. Tuberculosis in California, from July, 1894, to July, 1895, stands at the head of the death-list; 1789 being recorded from this cause out of a total death-list in the State of 11,349. Due credit was given the veterinarian at a meeting in San José for enlightening the public on this all-important question.

Veterinarian Button is doing valuable missionary service through the public press of Riverside.

Veterinarian Pierce, of Oakland, besides officiating as milk-inspector, includes meat, market, and bakery inspection, treats all city horses, inspects all purchased, and also the hay purchased for the latter, and receives one hundred dollars per month for his services, out of which he must maintain a horse and carriage. Wake up, Californians! You have not as overworked or ill-paid an officer in your whole county's list of public servants.

San José and Santa Clara Counties, through the proper authorities, are taking active steps toward supporting the inspectors against cows infected with tuberculosis.

Los Angeles, through Veterinarian Blackington, city meat- and milk-inspector, is active in the warfare against impure and unwholesome food-products.

Warfare against unqualified milk- and meat-inspectors is being waged in San Francisco. It is surely needed, and every veterinarian in San Francisco should hold up Dr. Creely's hands in this crusade against ignorance.

The State authorities are being urged by the cities to take hold of the suppression of tuberculosis, through the Board of Health of that commonwealth, particularly in the line of breaking up the nefarious traffic in condemned and other diseased animals, as well as the prevention of transferring herds of animals condemned in one county to some other county, and the shipment of impure milk into the large cities. The San Francisco *Chronicle* demands the prosecution and punishment of those known to sell diseased meat in the city's markets, and calls upon the veterinary inspectors to lead the movement.

